# Efficacy of Biphasic Calcium Phosphate Ceramic With a Needle-Shaped Surface Topography Versus Autograft in Instrumented Posterolateral Spinal Fusion

**DOI:** 10.1097/BRS.0000000000005075

**Published:** 2024-06-17

**Authors:** Hilde W. Stempels, A. Mechteld Lehr, Diyar Delawi, Eric A. Hoebink, Inge A.A.A. Wiljouw, Diederik H.R. Kempen, Job L.C. van Susante, Moyo C. Kruyt

**Affiliations:** a Department of Orthopaedic Surgery, University Medical Center Utrecht, Utrecht, The Netherlands; b Department of Orthopaedic Surgery, St. Antonius Hospital, Utrecht, The Netherlands; c Department of Orthopaedic Surgery, Amphia Hospital, Breda, The Netherlands; d Department of Orthopaedic Surgery, OLVG, Amsterdam, The Netherlands; e Department of Orthopaedic Surgery, Rijnstate Hospital, Arnhem, The Netherlands

**Keywords:** Adult, autograft, biphasic calcium phosphate, bone graft substitute, intrapatient, noninferiority, posterolateral fusion, PLF, randomized controlled trial, spinal fusion, standalone

## Abstract

**Study Design.:**

A multicenter randomized controlled noninferiority trial with intrapatient comparisons.

**Objective.:**

The aim of this study was to determine noninferiority of a slowly resorbable biphasic calcium phosphate with submicron microporosity (BCP<μm, MagnetOs Granules) as an alternative for autograft in instrumented posterolateral fusion (PLF).

**Summary of Background Data.:**

Successful spinal fusion with a solid bone bridge between the vertebrae is traditionally achieved by grafting with autologous iliac bone. However, the disadvantages of autografts and unsatisfactory fusion rates have prompted the exploration of alternatives, including ceramics. Nevertheless, clinical evidence for the standalone use of these materials is limited.

**Methods.:**

Adults indicated for instrumented PLF (1 to 6 levels) were enrolled at 5 participating centers. After bilateral instrumentation and fusion-bed preparation, the randomized allocation side (left or right) was disclosed. Per segment 10 cc of BCP<μm granules (1 to 2 mm) were placed in the posterolateral gutter on one side and 10 cc autograft on the contralateral side. Fusion was systematically scored on 1-year follow-up CT scans. The study was powered to detect >15% inferiority with binomial paired comparisons of the fusion performance score per treatment side.

**Results.:**

Of the 100 patients (57 ± 12.9 y, 62% female), 91 subjects and 128 segments were analyzed. The overall posterolateral fusion rate per segment (left and/or right) was 83%. For the BCP<μm side only the fusion rate was 79% versus 47% for the autograft side (difference of 32 percentage points, 95% CI, 23-41). Analysis of the primary outcome confirmed the noninferiority of BCP<μm with an absolute difference in paired proportions of 39.6% (95% CI, 26.8-51.2; *p* < 0.001).

**Conclusion.:**

This clinical trial demonstrates noninferiority and indicates superiority of MagnetOs Granules as a standalone ceramic when compared to autograft for posterolateral spinal fusion. These results challange the belief that autologous bone is the most optimal graft material.

Successful spinal fusion relies on the formation of a solid bone bridge between the individual vertebrae. To achieve this, autologous iliac crest bone has been used since the beginning of spine surgery.^1^ Several disadvantages are known to exist, including, most importantly, the limited availability, extra surgical time, and donor-site morbidity. Although some studies have shown that pain is not increased when the iliac crest graft is harvested via the same incision.^2,3^

Another important concern is the relatively low fusion rate of autograft in PLF. Several clinical studies reported that at 1 year of follow-up, roughly half of unilateral PLFs grafted with autograft did not form a solid posterolateral fusion bridge.^4–7^ Although this deficiency may be largely mitigated by strong instrumentation and (facet) ankylosis at later time points, it also highlights an opportunity for improvement in bone grafting in PLF.^8^

Exciting technologies have been explored to provide a better substitute for autograft, such as bone morphogenetic proteins and cell-based strategies, but general acceptance and future medical registration are questionable.^7,9–11^ Much less controversial are allograft or ceramics, although their superiority as standalone alternative has not been demonstrated.^12^ Even noninferiority is not generally accepted and has only recently been demonstrated for specific ceramics.^13^

Ceramics offer numerous advantages as bone graft substitutes, including minimal disease transmission risk, excellent biocompatibility, long shelf life, and cost-effective manufacturing. As a consequence, ceramics have been used in spinal fusion procedures for decades and intensive research to improve their performance has continued.^14^ These investigations yielded insights into optimal material and surface compositions, resulting in biphasic calcium phosphate ceramics with submicron topography and microporosity, which have shown osteoinductivity and superior effectiveness in various preclinical models.^15–18^ The exact mechanism of the submicron surface topography remains elusive but is attributed to (mechanical) stimulation of macrophages, resulting in the induction of bone in animal models.^19–22^

Our research group previously investigated such a commercialized microporous biphasic calcium phosphate in a randomized clinical trial and demonstrated that the standalone use of this AttraX Putty (NuVasive Inc., CA) in PLF was noninferior to autograft.^13^ In that study, we also recognized rapid resorption of both the iliac crest autograft and ceramic within the first year, leaving only half of the intended fusions successful. We therefore seized the opportunity to investigate a modified version of this ceramic (MagnetOs Granules, Kuros Biosciences B.V., Bilthoven, The Netherlands; referred to as BCP<μm), designed with a slower resorption rate and a surface topography consisting of submicron needles instead of micrograins.

## MATERIALS AND METHODS

### Study Design

This study is a multicenter, randomized, intrapatient controlled noninferiority trial (ClinicalTrials.gov NCT03625544). The study design and protocol are similar to a previous trial recently published by our research group and discussed elsewhere.^13,23^

After obtaining approval by the medical research ethics committee of the University Medical Center Utrecht and local institutional review boards, the study was conducted in 5 Dutch hospitals, in accordance with international legislation and Dutch law. Based on computerized simple randomization, each subject got one side of their spinal fusion trajectory grafted with the BCP<μm ceramic and the contralateral side treated with autograft. At 1-year follow-up, the primary efficacy outcome was assessed on CT scans to evaluate posterolateral fusion. Fusion performance of the BCP<μm was tested with a noninferiority margin of 15%. Safety was evaluated by analysis of (serious) adverse events.

### Subjects

Patients between 18 and 80 years of age undergoing primary instrumented posterolateral spinal fusion of 1 to 6 levels in the thoracolumbar region were considered eligible for this study. The complete list of inclusion and exclusion criteria can be found in Table 1.

**TABLE 1:** Inclusion and Exclusion Criteria

Inclusion criteria
1. Instrumented posterolateral thoracolumbar spinal fusion, with or without additional posteriorly inserted interbody devices (PLIF, TLIF), because of deformity*, structural instability† and/or expected instability‡
2. Non-responsive to ≥6 mo of nonoperative treatment
3. Fusion indicated for 1 to 6 levels in the T10 to S2 region. In case of extensive osteotomies (PSO or VCR) the osteotomized segment will not be included in the assessment
4. Skeletally mature, between 18 and 80 y old
5. Informed consent
Exclusion criteria
1. Previous surgical attempt(s) for fusion of the intended segment(s)
2. Previous treatments that compromise fusion surgery
3. Previous autologous bone harvesting that compromise the quality and amount of iliac crest bone grafting
4. Indication for spinal fusion because of an acute traumatic reason
5. Active spinal and/or systemic infection
6. Spinal metastasis in the area intended for fusion
7. Systemic disease or condition affecting the ability to participate in the study
8. Risk for noncompliance
9. Participation in clinical trials evaluating investigational devices, pharmaceuticals or biologics <3 mo of enrollment
10. Intended pregnancy <1.5 y of enrollment
11. Body mass index >36
12. Expected to require additional surgery to the same spinal region <6 mo
13. Current or recent (<1 y) corticosteroid use equivalent to prednisone ≥5 mg/day, prescribed for >6 wk

* Deformity is defined as a scoliosis in the coronal plane of >20° and/or a sagittal balance disturbance according the SRS/Schwab classification on standardized standing full spine radiographs.

† Preoperative instability is defined as a progressive angular deformity or spondylolisthesis in standing radiographs.

‡ Spinal stenosis is based on radiological and clinical findings.

### Investigational Product

**Figure 1:**
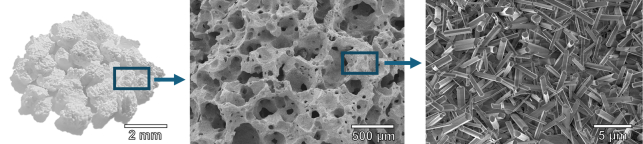
The BCP<µm granules of 1 to 2 mm in size have a macroporosity and microporosity and display a characteristic submicron needle-shaped surface topography when observed at high magnifications with a scanning electron microscope.

MagnetOs Granules comprises a biphasic calcium phosphate ceramic with 65% to 75% Tri-Calcium Phosphate (TCP—Ca_3_(PO_4_)_2_) and 25% to 35% Hydroxyapatite (HA—Ca_10_(PO_4_)_6_ (OH)_2_) with a total porosity of 70 ± 15% and pore diameter range of 0 to 1000 µm. The granules (1 to 2 mm in size) undergo a hydrothermal treatment (i.e., autoclaving) resulting in the submicron needle-shaped surface topography (Fig. 1).

### Surgical Technique

All subjects underwent a single or multilevel PLF with pedicle screw instrumentation through a midline approach. When deemed necessary, decompression and/or an additional interbody fusion procedure with local bone graft were performed. After placement of instrumentation and bilateral fusion-bed preparation via decortication, the randomized allocation side (left/right) of the BCP<μm condition was revealed by opening a sealed envelope.

For each segment, 10 cc of the BCP<μm granules were prepared in a surgical steel bowl by soaking them in 10 mL venous blood that was allowed to clot. The resulting slurry was then positioned onto the graft bed with a 20 cc syringe.

For autograft, corticocancellous bone was harvested from the posterior iliac crest on the autograft allocation side, through the initial skin incision. Both local decompression bone and iliac crest bone were morselized into 2 to 4 mm pieces. To match the contralateral use of 10 cc of BCP<μm, a volume of 8 to 10 cc autograft per fusion level was intended. The contribution of iliac crest bone to the autograft condition had to be at least 50%. Graft volumes were assessed by slight compression in a 20 cc syringe, that was then used to position the graft.

Both grafts were placed at the allocated side around the posterior instrumentation in the decorticated lateral gutters, bridging the dorsal surfaces of the transverse processes, facets, and laminae. The wound was then closed in layers, followed by standard postoperative care.

### Outcome Measures

Clinical and radiographic assessments were conducted preoperatively, and at 6 weeks, 3 months, and 1 year postoperatively. Patient-reported outcomes measures (PROMs) included a Visual Analogue Scale (VAS) for back and leg pain, the Oswestry Disability Index (ODI) and the EQ-5D-5L. The condition-specific ODI ranges from 0% to 100%, with higher scores indicating greater functional disability related to low back pain.^24^ A minimal clinically important difference (MCID) of 15 points was applied to the VAS and ODI and a ODI score of ≤22% was considered a satisfactory symptom state.^25–27^ Generic health status was measured with the EQ-5D-5L and converted into a single index value ranging from -0.446 (worst health state) to 1.000 (full health).^28^

### Fusion Assessment

For the primary efficacy outcome, thin-sliced (≤1 mm) CT scans with multiplanar reconstructions were obtained at the 1-year follow-up. Posterolateral fusion was evaluated independently by 2 spine surgeons blinded to the treatment sides using the previously developed assessment method based on Christensen (2001) and Carreon (2007).^8,29,30^ Interobserver reliability of this method is moderate (Kappa = 0.45) and comparable to other radiological studies.^13^ Both sides of each instrumented segment were evaluated in 3 reconstructed planes. To discriminate ceramic remnants from bone, the scatter reduction was switched of. The intertransverse area and the area around the rod, including the facet joint, were scored separately as fusion, doubtful fusion or nonunion. Additional interbody fusion was assessed similarly in the sagittal and coronal planes. CT scans with disagreements were re-examined to reach consensus. For statistical analyses, the posterolateral fusion scores of each segment and side, as well as the scores for interbody fusion, were dichotomized into “fused” (fusion) and “not fused” (doubtful fusion or nonunion).

### Safety Evaluation

To assess safety, adverse events potentially associated with the (surgical) procedure were recorded until last follow-up and examined for any potential relation with BCP<μm. Adverse events were defined as any unexpected, undesirable medical experience occurring to a subject during the study. Events were classified as serious when they resulted in death, were life-threating, required hospitalization or prolongation of existing hospitalization, and/or resulted in persistent or significant disability or incapacity.

### Statistical Methods

This study was powered based on an estimated unilateral fusion rate of 50% and 70% concordance between both sides of the fusion trajectory.^5,31–33^ Weighing the disadvantages of autografting against the consequences of less successful fusions at the BCP<μm side, the noninferiority margin was set at an absolute difference of 15%. With a desired power of 80% and one-sided significance level of 0.05, a minimum sample size of 84 patients was calculated. Assuming that ~15% of the subjects would not be evaluable for primary efficacy analysis (e.g., because of revision surgery with graft removal or lost to follow-up), the total number was set at 100.

Study data were processed in an electronic data capture system (Castor EDC, Ciwit BV, Amsterdam, The Netherlands) and analyzed using SPSS Statistics, version 29.0.1 (IBM Corp., New York, NY). Baseline characteristics, surgical details, PROMs and fusion rates on segment level were summarized using descriptive statistics. The VAS for back and leg pain and ODI at baseline and 1-year follow-up were compared with paired samples *t* test when applicable (*p* < 0.05).

To examine fusion at segment level while accounting for clustering of fusion scores within segments and within patients, a 3-level Generalized Estimating Equations (GEE) model with an independent correlation structure and treatment condition as predictor was used. The relation between successful interbody fusion and posterolateral fusion on either or both sides was analyzed using a similar 2-level GEE model with spinal level and interbody fusion as predictors. For both models, the significance level was *p* = 0.05 and the odds ratio (OR) along with the 95% confidence interval (CI) is reported.

For the primary outcome analyses, a posterolateral fusion performance score per treatment condition was calculated to correct for multilevel fusions. This score was based on a higher, equal or lower number of fused segments on one side compared to the contralateral side. That way each subject had a single outcome for each condition (1 = more or equal number of segments fused, 0 = less or none of the segments fused). Noninferiority of BCP<μm versus autograft was tested against the upper limit of the 2-sided 95% CI around the difference in paired proportions for successful posterolateral fusion performance, corresponding to a one-sided significance level of 0.025.

## RESULTS

### Patient Characteristics

**Figure 2:**
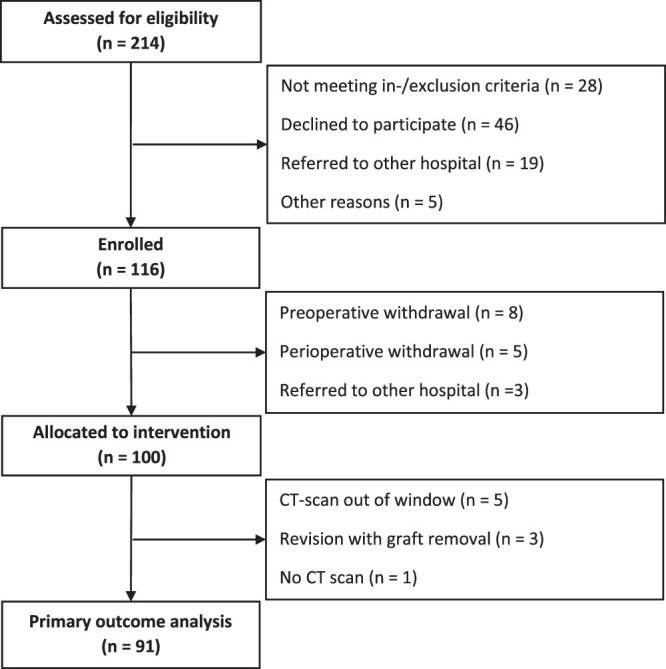
Flowchart of study enrollment and sample retention.

Between September 2018 and October 2022, 116 patients provided informed consent, of which 100 subjects were operated according to the randomization scheme. For the primary outcome analysis, 9 subjects were excluded for the circumstances outlined in Figure 2. Patient characteristics and surgical details are summarized in Table 2. The average age was 57 ± 12.9 (range 20 to 79) years, with 62% female. A total of 19 patients were active smokers, the rest were either former smokers (n = 35) or had never smoked (n = 46). The majority underwent surgery in the lumbosacral region (n = 54) and had a single-level fusion (n = 69). A total of 153 instrumented segments were involved, with 55 additional interbody procedures performed in 49 subjects.

**TABLE 2:** Patient Characteristics and Surgical Details (n = 100)

Age, mean ± SD (range), y	57.2 ± 12.9 (20–79)	
Sex, n (%)		
Male	38 (38%)	
Female	62 (62%)	
BMI, mean ± SD (range)	27.3 ± 4.1 (17.7–36.3)	
Smoking n (%)		
Nonsmoker	46 (46%)	
Ex-smoker	35 (35%)	
Smoker	19 (19%)	
Indication for instrumentation, n (%)^*^		
Deformity	49 (49%)	
Structural instability	29 (29%)	
Expected instability	23 (23%)	
ASA classification, n (%)		
I	20 (20%)	
II	61 (61%)	
III	19 (19%)	
Numbers of segments fused, n (%)		
1	69 (69%)	
2	19 (19%)	
>2	12 (12%)	
Median number of segments fused (range)	1 (1–5)	
Spinal region fused, n (%)		
Thoracolumbar	4 (4%)	
Lumbar	42 (42%)	
Lumbosacral	54 (54%)	
Decompression, n (%)	94 (94%)	
Interbody device, n (%)	49 (49%)	
Level and type of interbody device, n	PLIF	TLIF
L2-L3	0	2
L3-L4	5	2
L4-L5	18	6
L5-S1	19	3
Operative time, n (%)		
<2 h	10 (10%)	
2–4 h	83 (83%)	
>4 h	7 (7%)	
Blood loss, median (range), cc	500 (20–2200)	
Length of stay, median (range), d	3 (1–40)	

^*^ Subjects could have multiple indications for surgery.

n indicates number of subjects; PLIF, posterior lumbar interbody fusion; TLIF, transforaminal lumbar interbody fusion.

### Patient Reported Outcomes

**Figure 3:**
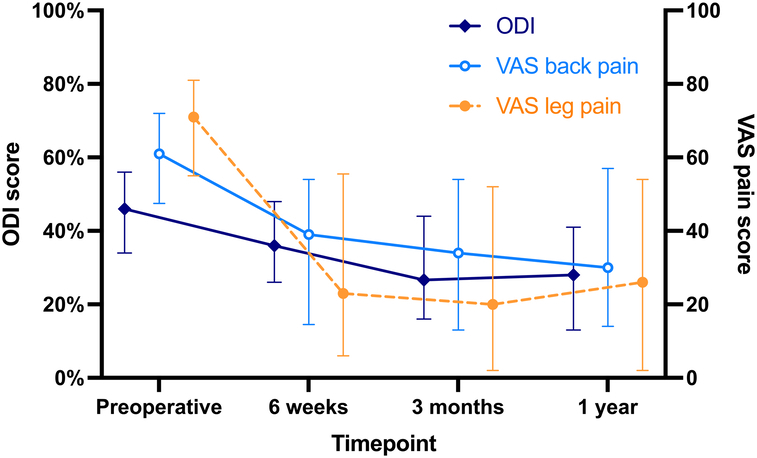
ODI (0%-100%; dark blue line) and VAS pain (0–100; back pain in light blue line and leg pain in orange dotted line) scores at baseline and each postoperative follow-up. Median values along with their interquartile range are given as the data are not normally distributed. ODI indicates Oswestry Disability Index; VAS, Visual Analogue Scale.

During the first year after surgery, clinical outcomes improved, with a mean decrease in ODI of 18 ± 16 percentage points and VAS scores of 24 ± 29 points for back pain (*p* < 0.001). The decrease in leg pain (median 33 points, interquartile range [IQR] 11 to 65) was not normally distributed and therefore not tested. This improvement is shown in Figure 3 and exceeded the MCID for the majority of patients (VAS back pain 62%, VAS leg pain 67% and ODI 59%). Furthermore, at 1-year follow-up 44% of the subjects achieved a satisfactory symptom state ≤22% on ODI. Improvement in clinical status is also reflected in the increased EQ-5D-5L index value, from median 0.40 (IQR 0.24 to 0.58) to median 0.77 (IQR 0.59 to 0.85).

### Fusion Assessment

Posterolateral and interbody fusion were assessed in 91 CT scans obtained at 1-year follow-up, encompassing 132 instrumented segments. Four segments were excluded; 2 due to noncompliance with the study procedure regarding grafting, and 2 because of a pedicle subtraction osteotomy.

Of the 128 segments assessed for posterolateral fusion, 83% were fused on either one or both sides. For the BCP<μm side, this was 79% vs. 47% for the autograft side (absolute difference of 32 percentage points, 95% CI, 23-41). The estimated odds ratio favored the BCP<um side at 4.2 (95% CI, 2.7-6.8). For 40% of segments with only one side fused, there is a sharp contrast in the number of fusions between BCP<μm (46 cases) and autograft (5 fusions) (Table 3).

**TABLE 3:** Posterolateral Fusion Per Treatment Condition Per Segment (n = 128)

	Autograft		
	Not fused	Fused	Total
BCP<μm			
Not fused	22	5	27
Fused	46	55	101
Total	68	60	128

Interbody fusions were assessed in 54 segments, of which 24 were fused. The overall segment fusion rate was a little higher (84%), as 2 levels with unsuccessful posterolateral fusion had successful interbody fusion. Segments with an interbody fusion procedure had a slightly lower overall posterolateral fusion rate compared with segments without (78% vs. 87%). Secondary GEE-analyses, however, showed a positive relation between successful interbody fusion and posterolateral fusion (OR = 5.5; 95% CI, 1.2 to 24.4; *p* = 0.025), which most likely represents patient specific factors.

The primary outcome is the fusion performance score per treatment condition that adjusts for multilevel fusions. This analysis confirmed the noninferiority of BCP<μm with an absolute difference in paired proportions of 39.6% (95% CI, 26.8-51.2; *p* < 0.001; Table 4), which even indicates the superiority of the BCP<μm.^34^

**TABLE 4:** Posterolateral Fusion Performance Per Treatment Condition, After Correction for Multilevel Fusion (n = 91)

	Fusion performance score autograft side		
	0	1	Total
Fusion performance score BCP<μm side			
0	17	5	22
1	41	28	69
Total	58	33	91

Performance score of 1 means more or an equal number of segments fused on that side compared with the contralateral side, 0 is less or none of the segments fused. The absolute difference in paired proportions of successful fusion performance was 39.6% with a 95% CI (26.8-51.2), *p* < 0.001.

### Safety Evaluation

During the first year, there were 24 serious adverse events related to the (surgical) procedure, involving 17 subjects and 14 reoperations (see Table 5). Reasons for reoperation included surgical site infection (n = 7), persistent cerebrospinal fluid leakage (n = 4), neurologic complications arising from a malpositioned screw (n = 1), and need for extension of the instrumentation (n = 2). Only 3 reoperations included graft removal. Overall, 36 adverse events were reported, including 10 cases of dural tears that were repaired before graft placement. None of the (serious) adverse events could be directly related to BCP<μm.

**TABLE 5:** Number and Nature of Serious Adverse Events (n = 100)

Surgical site infection	7
Pain treatment	5
Symptomatic dural tear	5
Neurologic complications	2
Prolonged wound leakage	2
Gastrointestinal complications	1
Instrumentation failure	1
Miscellaneous	1

## DISCUSSION

The current study builds upon previous work that investigated a comparable microporous biphasic calcium phosphate (AttraX Putty).^8,13,23^ These studies established the effectiveness of the intrapatient controlled design to compare bone graft substitutes to the gold standard, i.e. autologous bone. To avoid bone graft quality as a potential confounder when only local graft is used, we decided to use at least 50% iliac crest bone graft. Like other studies, we demonstrated that achieving fusion with autologous bone graft at a single intended graft location like the posterolateral gutter is challenging and does not exceed 55% after 1 year.^4,5,13^ The observation that most autograft and ceramics are resorbed within a year, but fusion continues thereafter,^8^ supports the idea that posterolateral fusion relies more on facet ankylosis than on graft-related bone bridge formation on the long term. Moreover, it suggests osteoconductive fusion is not optimally facilitated by autografts nor most ceramics. A lower resorption rate is a key difference between the microporous ceramic previously investigated and the BCP<μm investigated in the current trial.

Even though the primary aim of the current study was to demonstrate noninferiority, our findings indicate superiority of the BCP<μm in terms of CT determined posterolateral fusion at 1 year. This superiority became evident with the primary outcome, the fusion performance score, that adjusts for multilevel procedures by comparing one treatment side to the other. With the additional analysis (GEE), examining fusion rates per segment, we similarly observed the superiority of the BCP<μm condition. This was most prominent when looking at the unilaterally fused segments in Table 3, were BCP<μm was responsible for the fusion in 46 of 51 cases. We realize that the observed superiority of a standalone ceramic has not been shown before and definitely needs confirmation by others in future studies.

**Figure 4:**
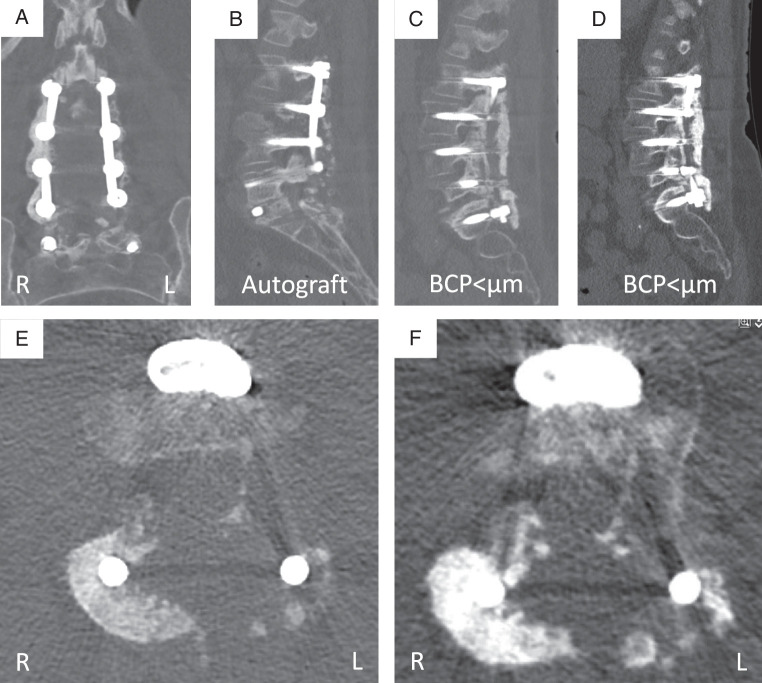
Computed tomography images of a 70-year-old female 1 year after L2-S1 PLF with L2-3 and L5-S1 TLIFs. (A) Coronal reconstruction showing dense ceramic granule remnants around the rod on the right side. (B) Sagittal reconstruction of the left side showing some autograft remnants posterior to the rod and no signs of fusion. (C) Sagittal reconstruction of the right side indicating bony fusion anterior to the rod and a dense mass posterior to the rod. (D) Without scatter reduction this mass clearly contains ceramic remnants. (E) Axial view L2-3 indicating a mass around the right rod, that can be identified as ceramic remnants without the scatter reduction (F).

**Figure 5:**
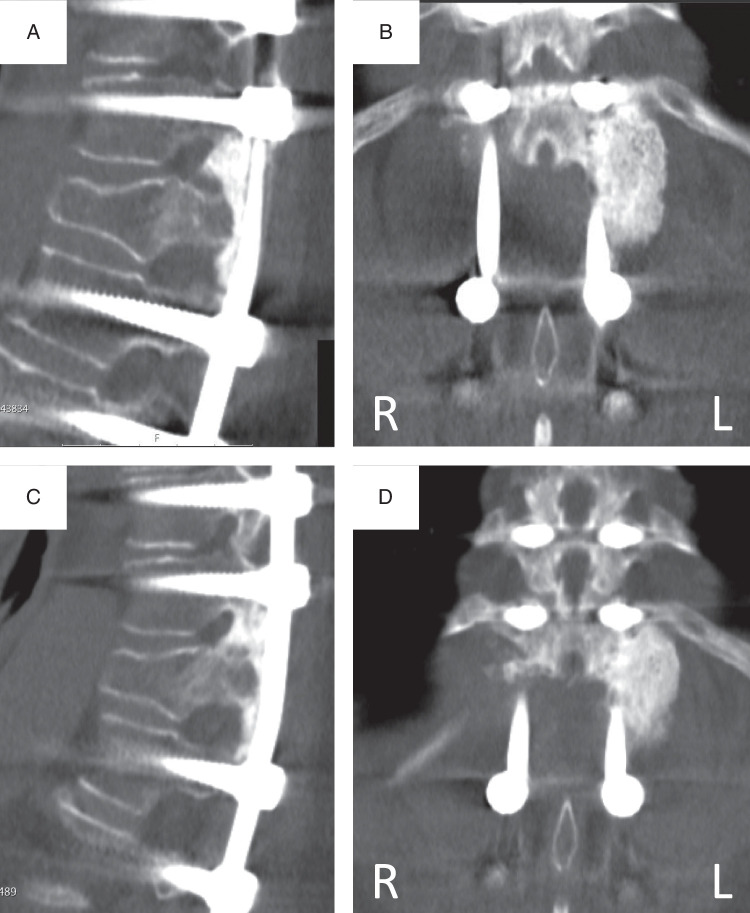
Sagittal and coronal computed tomography images of T12-L1 fusion area with BCP<µm placed on the left side, at 1 year (A and B) and 2 years (C and D) follow-up. One year after surgery the ceramic granules are distinct from bone, fusion was scored as doubtful. After 2 years the granules remodeled to a bony fusion.

There are some important limitations to the current study. First, we used an outcome measure that at best only indicates if the intended fusion has been achieved. Even if this leads to an improved clinical outcome after 1 year, the intrapatient model does not allow for comparison of patient reported outcomes. To really demonstrate clinical benefit, thousands of patients are needed, probably with a much longer follow-up. We have chosen the objective outcome of radiographic fusion as this is the purpose of the grafting procedure. Second, the intrapatient design only assessed unilateral fusion, which underestimates the fusion rate when any fusion (left and/or right) would be regarded as a fusion. Third, the reliability of the thin-slice CT assessment for fusion determination is not fully established, as highlighted in a recent systematic review and reflected by the moderate interobserver reliability.^35^ Fourth, because of the slower absorption rate of this BCP<μm, we are not completely sure that the fusion observed in this condition always represents bone and is not a remnant of BCP<μm that perfectly mimics bone. However, by adjusting the scatter reduction function, BCP<μm granular remnants could be identified in 1 year CT scans and distinguished from bone relatively easily (Fig. 4). Interestingly, in subsequent 2 year CT scans (not part of the current study) the granules appear to remodel into bone (Fig. 5). This reveals a fifth limitation, that in many cases the observers could not be truly blinded. Given the radiological resemblances between ceramics and bone, it will be very difficult to completely exclude the human factor for this assessment. Conducting fusion assessments after a longer follow-up period would afford the BCP<μm more time to dissolve, but reduces the graft related component of fusion.

## CONCLUSIONS

This clinical trial demonstrates the noninferiority and potential superiority of BCP<μm compared to autologous bone graft in terms of CT-assessed posterolateral spinal fusion after 1 year. Therefore, MagnetOs™ Granules could serve as a standalone bone graft substitute for autograft in instrumented thoracolumbar PLF.
